# Pregnancy, Delivery, and Neonatal Outcomes Associated With Maternal Obsessive-Compulsive Disorder: Two Cohort Studies in Sweden and British Columbia, Canada

**DOI:** 10.1001/jamanetworkopen.2023.18212

**Published:** 2023-06-14

**Authors:** Lorena Fernández de la Cruz, K. S. Joseph, Qi Wen, Olof Stephansson, David Mataix-Cols, Neda Razaz

**Affiliations:** 1 Centre for Psychiatry Research, Department of Clinical Neuroscience, Karolinska Institutet, Stockholm, Sweden; 2 Stockholm Health Care Services, Region Stockholm, Stockholm, Sweden; 3 Department of Obstetrics and Gynaecology, University of British Columbia and the Children’s and Women’s Hospital and Health Centre of British Columbia, Vancouver, British Columbia, Canada; 4 School of Population and Public Health, University of British Columbia, Vancouver, British Columbia, Canada; 5 Department of Women’s Health, Karolinska University Hospital, Stockholm, Sweden; 6 Clinical Epidemiology Division, Department of Medicine Solna, Karolinska Institutet, Stockholm, Sweden; 7 Department of Clinical Sciences, Lund University, Lund, Sweden

## Abstract

**Importance:**

Obsessive-compulsive disorder (OCD) is associated with adverse health-related outcomes. However, pregnancy and neonatal outcomes among women with OCD have been sparsely studied.

**Objective:**

To evaluate associations of maternal OCD with pregnancy, delivery, and neonatal outcomes.

**Design, Setting, and Participants:**

Two register-based cohort studies in Sweden and British Columbia (BC), Canada, included all singleton births at 22 weeks or more of gestation between January 1, 1999 (Sweden), or April 1, 2000 (BC), and December 31, 2019. Statistical analyses were conducted between August 1, 2022, and February 14, 2023.

**Exposure:**

Maternal OCD diagnosis recorded before childbirth and use of serotonin reuptake inhibitors (SRIs) during pregnancy.

**Main Outcomes and Measures:**

Pregnancy and delivery outcomes examined were gestational diabetes, preeclampsia, maternal infection, antepartum hemorrhage or placental abruption, premature rupture of membranes, induction of labor, mode of delivery, and postpartum hemorrhage. Neonatal outcomes included perinatal death, preterm birth, small for gestational age, low birth weight (<2500 g), low 5-minute Apgar score, neonatal hypoglycemia, neonatal jaundice, neonatal respiratory distress, neonatal infections, and congenital malformations. Multivariable Poisson log-linear regressions estimated crude and adjusted risk ratios (aRRs). In the Swedish cohort, sister and cousin analyses were performed to account for familial confounding.

**Results:**

In the Swedish cohort, 8312 pregnancies in women with OCD (mean [SD] age at delivery, 30.2 [5.1] years) were compared with 2 137 348 pregnancies in unexposed women (mean [SD] age at delivery, 30.2 [5.1] years). In the BC cohort, 2341 pregnancies in women with OCD (mean [SD] age at delivery, 31.0 [5.4] years) were compared with 821 759 pregnancies in unexposed women (mean [SD] age at delivery, 31.3 [5.5] years). In Sweden, maternal OCD was associated with increased risks of gestational diabetes (aRR, 1.40; 95% CI, 1.19-1.65) and elective cesarean delivery (aRR, 1.39; 95% CI, 1.30-1.49), as well as preeclampsia (aRR, 1.14; 95% CI, 1.01-1.29), induction of labor (aRR, 1.12; 95% CI, 1.06-1.18), emergency cesarean delivery (aRR, 1.16; 95% CI, 1.08-1.25), and postpartum hemorrhage (aRR, 1.13; 95% CI, 1.04-1.22). In BC, only emergency cesarean delivery (aRR, 1.15; 95% CI, 1.01-1.31) and antepartum hemorrhage or placental abruption (aRR, 1.48; 95% CI, 1.03-2.14) were associated with significantly higher risk. In both cohorts, offspring of women with OCD were at elevated risk of low Apgar score at 5 minutes (Sweden: aRR, 1.62; 95% CI, 1.42-1.85; BC: aRR, 2.30; 95% CI, 1.74-3.04), as well as preterm birth (Sweden: aRR, 1.33; 95% CI, 1.21-1.45; BC: aRR, 1.58; 95% CI, 1.32-1.87), low birth weight (Sweden: aRR, 1.28; 95% CI, 1.14-1.44; BC: aRR, 1.40; 95% CI, 1.07-1.82), and neonatal respiratory distress (Sweden: aRR, 1.63; 95% CI, 1.49-1.79; BC: aRR, 1.47; 95% CI, 1.20-1.80). Women with OCD taking SRIs during pregnancy had an overall increased risk of these outcomes, compared with those not taking SRIs. However, women with OCD not taking SRIs still had increased risks compared with women without OCD. Sister and cousin analyses showed that at least some of the associations were not influenced by familial confounding.

**Conclusion and Relevance:**

These cohort studies suggest that maternal OCD was associated with an increased risk of adverse pregnancy, delivery, and neonatal outcomes. Improved collaboration between psychiatry and obstetric services and improved maternal and neonatal care for women with OCD and their children is warranted.

## Key Points

**Question:**

Is maternal obsessive-compulsive disorder (OCD) associated with adverse pregnancy, delivery, and neonatal outcomes?

**Findings:**

In 2 cohort studies including 10 653 pregnancies to mothers with OCD in Sweden and British Columbia, Canada, maternal OCD was associated with statistically significantly increased risk of adverse pregnancy, delivery, and neonatal outcomes. Sister and cousin analyses showed that at least some of the described associations were independent from familial confounding.

**Meaning:**

This study suggests that greater collaboration between psychiatrists and obstetricians and close monitoring of pregnant women with OCD and their newborns is warranted.

## Teaser

This cohort study evaluates associations of maternal obsessive-compulsive disorder (OCD) with pregnancy, delivery, and neonatal outcomes.

## Introduction

Obsessive-compulsive disorder (OCD) is a psychiatric disorder affecting 1% to 3% of the population.^1,2^ It is thought to be 1.6 times more likely in women than men.^2^ Obsessive-compulsive disorder is associated with an increased risk of various health-related issues.^3^ However, adverse pregnancy and neonatal outcomes in women with OCD have been seldom examined, to our knowledge.^4,5,6,7,8^ Furthermore, available studies have generally had methodological limitations, including small sample sizes^4,5^ or lack of appropriate comparison groups.^6,7^ Only one cohort study has explored intrapartum, delivery, and postpartum complications associated with OCD (3365 births to women with the disorder).^8^ In that investigation, maternal OCD was associated with higher risks of gestational hypertension, preeclampsia, premature rupture of membranes, instrumental and cesarean deliveries, venous thromboembolism, and preterm birth.

Most individuals with OCD in Sweden take medication for their symptoms, generally serotonin reuptake inhibitors (SRIs).^9^ However, the association of such medication with perinatal and neonatal outcomes has not been explored, to our knowledge. This is an important question because selective serotonin reuptake inhibitors (SSRIs) have been previously associated with adverse pregnancy and neonatal outcomes.^10,11^ In addition, familial confounding (ie, due to unmeasured shared genetic and environmental factors) could be masking a true association. We are unaware of any studies using sibling comparison analyses to provide insight into the role of shared familial factors in the association of maternal OCD with pregnancy, delivery, and neonatal outcomes.

We investigated pregnancy, delivery, and neonatal outcomes among births to women with OCD compared with births to women without OCD in 2 different population-based cohorts from Sweden and British Columbia (BC), Canada. We also examined differences in outcomes in mothers with OCD who were prescribed SRIs during pregnancy vs mothers with OCD not prescribed SRIs during pregnancy. In the Swedish cohort, familial confounding was explored using sister and cousin analyses.

## Methods

The study was approved by the Swedish Ethical Review Authority and the Clinical Research Ethics Board at the University of British Columbia. Informed consent was waived due to the register-based nature of the study and because participants were not identifiable. We followed the Strengthening the Reporting of Observational Studies in Epidemiology (STROBE) reporting guideline.

### Data Sources

The study comprised population-based cohorts from Sweden and the Canadian province of BC. Data from different population registers within each location were linked using person-unique identifiers.^12,13^

#### Swedish Data Sources

The Swedish Medical Birth Register contains prospectively recorded information since 1973 on antenatal, obstetric, and neonatal care for more than 98% of all births in Sweden.^14^ The National Patient Register (NPR) includes information on inpatient care for somatic and psychiatric disorders, with complete national coverage from 1987 and outpatient specialist services since 2001. Diagnoses in the NPR are based on the *International Classification of Diseases, Ninth Revision* (*ICD-9*: 1987-1996) and the *International Statistical Classification of Diseases and Related Health Problems, Tenth Revision* (*ICD-10*: 1997 and onward).^15^ The Prescribed Drug Register contains data on all dispensed prescriptions of medication (classified according to the Anatomical Therapeutic Chemical [ATC] system) to the whole population since July 1, 2005.^16^ The Multi-Generation Register connects every person born in Sweden since 1932, and ever registered as living in the country from 1961, with their biological or adoptive parents; this allows for the identification of relatives.^17^ The Total Population Register and the Education Register were used to extract sociodemographic information.

#### BC Data Sources

In BC, the provincial public health insurance program covers all eligible BC residents.^18^ Information about mothers, fetuses, and infants was obtained from the BC Perinatal Database Registry (BCPDR).^19^ This province-wide database includes information on antenatal, intrapartum and delivery, and postpartum maternal and infant care and outcomes for approximately 99% of births in BC since April 1, 2000. Validation studies show that the BCPDR is an accurate and comprehensive source of perinatal information.^20^ Other registers included the Discharge Abstract Database,^21^ which documents all BC hospital stays; the Medical Services Plan database,^22^ which includes all BC medical visits; the PharmaNet database,^23^ with information on dispensed prescriptions since 1995; and the Central Demographics File (previously the BC Consolidation file),^24^ detailing demographic and registration data on provincial health coverage. In BC, *ICD-9* codes were used from April 1, 1990, until 2000, and *ICD-10* codes have been used since 2001.

### Study Cohorts

Both cohorts included all singleton live births and stillbirths at 22 weeks or more of gestation between January 1, 1999, in Sweden, or April 1, 2000, in BC, and December 31, 2019. Mothers or offspring with missing IDs and offspring with missing sex were excluded. In addition, in the Swedish cohort, we identified the unaffected sisters of the mothers with OCD and their offspring (ie, cousins).

### Exposures

#### Maternal OCD

In both cohorts, women with an *ICD-9* or *ICD-10* diagnosis of OCD, recorded at least once between the age of 6 years (to avoid diagnostic misclassification and in line with previous register-based studies)^25,26^ and the index childbirth, were considered exposed. The validity and reliability of *ICD* codes for OCD in the Swedish NPR is high.^27^ The codes have not been validated in BC.

#### Maternal Use of SRIs

The recommended pharmacologic treatment for OCD is SRIs (ie, SSRIs or clomipramine).^28,29^ Using information from the Swedish Prescribed Drug Registry and the BC PharmaNet register, we defined exposure to these drugs by using ATC codes N06AB for SSRIs and N06AA04 for clomipramine. We identified the subcohorts of women who were dispensed an SRI in the period 30 days before the estimated day of conception to the day of the index childbirth during the period when dispensation data were available (ie, from July 2005 [in Sweden] and from 2000 [in BC] to 2019).

### Outcomes

Pregnancy and delivery outcomes examined in this study were gestational diabetes, preeclampsia, maternal infection, antepartum hemorrhage or placental abruption, premature rupture of membranes, induction of labor, mode of delivery, and postpartum hemorrhage. Neonatal outcomes included perinatal death (including stillbirths and neonatal deaths within the first 27 completed days after birth), preterm birth, small for gestational age, low birth weight (<2500 g), low 5-minute Apgar score (scores 4-6), neonatal hypoglycemia, neonatal jaundice, neonatal respiratory distress, neonatal infections, and congenital malformations detected during the first year after birth (divided into major and minor malformations). Specific *ICD* codes and definitions of outcomes are listed in eTables 1 and 2 in Supplement 1.

### Covariates and Additional Variables

Maternal characteristics examined included age at delivery, parity, place of birth, educational level (Swedish cohort only), cohabitation with a partner, height, body mass index (BMI), prepregnancy diabetes, prepregnancy hypertension, smoking during pregnancy, and year of delivery. We also studied maternal psychiatric comorbidity, including bipolar and psychotic disorders, and mood and anxiety disorders. A composite variable including any such psychiatric comorbidity was also examined. See eTables 1 and 2 in Supplement 1 for definitions and *ICD* codes.

### Statistical Analysis

Statistical analysis was performed from August 1, 2022, to February 14, 2023. We assessed the distribution of maternal characteristics by OCD status in both cohorts. Multivariable Poisson log-linear regression models were used to estimate unadjusted and adjusted risk ratios (aRRs). The main model adjusted for age at delivery, parity, place of birth, educational level, cohabitation with a partner, BMI, prepregnancy diabetes, smoking during pregnancy, and year of delivery. Confounders were included based on prior literature^8^ or statistical significance. For the primary research question, 3 additional models were used to adjust for maternal psychiatric comorbidity. The first model adjusted for the variables in the main analysis plus bipolar and psychotic disorders, the second model adjusted for the variables in the main analysis plus mood and anxiety disorders, and the third model adjusted for the variables in the main analysis and for any psychiatric comorbidity. Pregnancy and neonatal outcomes in women with OCD treated with SRIs during pregnancy (vs women with OCD not taking SRIs during pregnancy) were examined using the same methods mentioned. In a supplementary analysis, women with OCD not taking SRIs during pregnancy were also compared with unaffected women (only in the Swedish cohort due to the larger number of study individuals). As clustered robust SEs (to account for sequential births to the same mother) only changed 95% CIs in the third decimals, 95% CIs are reported with normal SEs without adjustment for clustering. Furthermore, to examine whether the association between maternal OCD and the outcomes was constant over time, we performed stratification according to calendar birth year. In the Swedish cohort, outcomes of OCD-exposed mothers and their children were compared with those of their non-OCD full sisters and their children (ie, offspring’s cousins) using logistic regression to estimate adjusted odds ratios (aORs) and corresponding 95% CIs. By design, this comparison controls for potential familial confounders that are shared by full siblings or cousins, including about 50% or 12.5% of the genetic make-up, respectively, and unmeasured shared environmental factors, such as socioeconomic status.^30^

Data management and analysis were performed using SAS, version 9.4 (SAS Institute Inc). All tests used 2-tailed significance set at *P* < .05. Given the exploratory nature of the study and the fact that the outcomes are interrelated, no adjustments for multiple comparisons were made.

## Results

### Association of Maternal OCD With Pregnancy, Delivery, and Neonatal Outcomes

The Swedish cohort included 2 145 660 pregnancies, of which 8312 (3.9 per 1000) pregnancies were to women with OCD (mean [SD] age at delivery, 30.2 [5.1] years), while the BC cohort included 824 100 pregnancies, of which 2341 (2.8 per 1000) pregnancies were to women with OCD (mean [SD] age at delivery, 31.0 [5.4] years) (Table 1). Women with OCD were more likely to be born in a Nordic country (Swedish cohort) or in Canada (BC cohort), have a lower educational level, live alone, be obese, smoke, and have higher rates of psychiatric comorbidity. A total of 6009 women with OCD (72.3%) in the Swedish cohort and 1184 women with OCD (50.6%) in the BC cohort had a diagnosis of any (other) psychiatric disorder, compared with 151 141 women without OCD (7.1%) in the Swedish cohort and 57 531 women without OCD (7.0%) in the BC cohort. Maternal OCD rates increased throughout the study period in both cohorts.

**Table 1.** Distribution of Maternal and Pregnancy Characteristics by Maternal OCD Status in Live Singleton Births in Sweden (1999-2019) and British Columbia, Canada (2000-2019)

Characteristic	No. (%)			
	Sweden (N = 2 145 660)		British Columbia, Canada (N = 824 100)	
	Mothers without OCD (n = 2 137 348)	Mothers with OCD (n = 8312)	Mothers without OCD (n = 821 759)	Mothers with OCD (n = 2341)
Age at delivery, y				
≤19	30 582 (1.4)	99 (1.2)	13 975 (1.7)	37 (1.6)
20-24	264 955 (12.4)	1040 (12.5)	85 881 (10.5)	263 (11.2)
25-29	658 370 (30.8)	2593 (31.2)	198 598 (24.2)	581 (24.8)
30-34	737 912 (34.5)	2832 (34.1)	286 961 (34.9)	828 (35.4)
≥35	445 529 (20.8)	1748 (21.0)	236 341 (28.8)	632 (27.0)
Missing	0	0	≤5	≤5
Parity				
1	939 325 (43.9)	3901 (46.9)	384 029 (46.7)	1060 (45.3)
2	780 454 (36.5)	2872 (34.6)	295 614 (36.0)	821 (35.1)
3	289 075 (13.5)	1055 (12.7)	98 194 (11.9)	310 (13.2)
≥4	128 494 (6.0)	484 (5.8)	43 850 (5.3)	150 (6.4)
Missing	0	0	72 (0.009)	≤5
Place of birth^a^				
Nordic country	1 669 074 (78.1)	7631 (91.8)	NA	NA
Non-Nordic country	465 942 (21.8)	672 (8.1)	NA	NA
Missing	2332 (0.1)	9 (0.1)	NA	NA
Place of birth^a^				
Canada	NA	NA	527 722 (64.2)	2002 (85.5)
Asia	NA	NA	182 396 (22.2)	112 (4.8)
Europe	NA	NA	37 781 (4.6)	85 (3.6)
Other	NA	NA	33 300 (4.1)	59 (2.5)
Missing	NA	NA	40 560 (4.9)	83 (3.5)
Education, y^b^				
≤9	181 108 (8.5)	1123 (13.5)	NA	NA
10-11	237 646 (11.1)	885 (10.6)	NA	NA
12	550 278 (25.7)	2367 (28.5)	NA	NA
13-14	305 907 (14.3)	1126 (13.5)	NA	NA
≥15	841 857 (39.4)	2785 (33.5)	NA	NA
Missing	20 552 (1.0)	26 (0.3)	NA	NA
Cohabitation with a partner				
Yes	1 908 368 (89.3)	6995 (84.2)	572 488 (69.7)	1399 (59.8)
No	123 747 (5.8)	901 (10.8)	214 983 (26.2)	856 (36.6)
Missing	105 233 (4.9)	416 (5.0)	34 288 (4.2)	86 (3.7)
Maternal height, cm				
≤159	294 326 (13.8)	1002 (12.1)	153 957 (18.7)	342 (14.6)
160-164	540 319 (25.3)	2096 (25.2)	183 227 (22.3)	508 (21.7)
165-169	606 836 (28.4)	2467 (29.7)	167 945 (20.4)	548 (23.4)
≥170	657 498 (30.8)	2636 (31.7)	167 661 (20.4)	564 (24.1)
Missing	38 369 (1.8)	111 (1.3)	148 969 (18.1)	379 (16.2)
Maternal BMI^c^				
<18.5	48 409 (2.3)	264 (3.2)	34 377 (4.2)	83 (3.5)
18.5-24.9	1 174 938 (55.0)	4341 (52.2)	360 681 (43.9)	976 (41.7)
25.0-29.9	495 237 (23.2)	1960 (23.6)	127 030 (15.5)	379 (16.2)
30.0-34.9	171 610 (8.0)	756 (9.1)	47 559 (5.8)	186 (7.9)
35.0-39.9	53 699 (2.5)	310 (3.7)	18 893 (2.3)	92 (3.9)
≥40.0	19 051 (0.9)	119 (1.4)	10 277 (1.3)	64 (2.7)
Missing	174 404 (8.2)	562 (6.8)	222 942 (27.1)	561 (24.0)
Prepregnancy diabetes				
Yes	28 119 (1.3)	151 (1.8)	4238 (0.5)	21 (0.9)
No	2 109 229 (98.7)	8161 (98.2)	817 521 (99.5)	2320 (99.1)
Prepregnancy hypertension				
Yes	14 754 (0.7)	63 (0.8)	4754 (0.6)	22 (0.9)
No	2 122 594 (99.3)	8249 (99.2)	817 005 (99.4)	2319 (99.1)
Smoking during pregnancy				
Yes	158 426 (7.4)	918 (11.0)	71 337 (8.7)	308 (13.2)
No	1 897 153 (88.8)	7147 (86.0)	286 565 (34.9)	925 (39.5)
Missing	81 769 (3.8)	247 (3.0)	463 857 (56.4)	1108 (47.3)
Year of delivery				
1999-2002^d^	345 596 (16.2)	120 (1.4)	107 880 (13.1)	113 (4.8)
2003-2007	488 340 (22.8)	631 (7.6)	200 502 (24.4)	337 (14.4)
2008-2012	531 694 (24.9)	2067 (24.9)	214 078 (26.1)	651 (27.8)
2013-2019	771 718 (36.1)	5494 (66.1)	299 299 (36.4)	1240 (53.0)
Psychiatric comorbidities^e^				
Any psychiatric disorder	151 141 (7.1)	6009 (72.3)	57 531 (7.0)	1184 (50.6)
Bipolar and psychotic disorders	13 751 (0.6)	980 (11.8)	7612 (0.9)	269 (11.5)
Mood and anxiety disorders	147 225 (6.9)	5940 (71.5)	54 923 (6.7)	1155 (49.3)

Abbreviations: BMI, body mass index (calculated as weight in kilograms divided by height in meters squared); NA, not applicable; OCD, obsessive-compulsive disorder.

^a^ Mother’s place of birth was categorized differently in the 2 cohorts.

^b^ Information on education was not available for the British Columbia cohort.

^c^ In Sweden, refers to early pregnancy BMI; in British Columbia, refers to prepregnancy BMI.

^d^ For the British Columbia cohort, this bracket includes deliveries for the period from 2000 to 2002 only.

^e^ *Any psychiatric disorder* includes bipolar and psychotic disorders and/or mood and anxiety disorders; *bipolar and psychotic disorders* include a record of a manic episode or bipolar disorder, or a record of schizophrenia or other psychotic disorders; *mood and anxiety disorders* include a record of depression or other mood disorders, or a record of phobic, anxiety, reaction to severe stress, or adjustment disorders (see *ICD* codes in eTable 1 in Supplement 1).

Rates and unadjusted analyses showing crude associations between maternal OCD and outcomes in Sweden and BC are shown in Table 2. Adjusted analyses showed that, in Sweden, maternal OCD was associated with 40% increased risk of gestational diabetes (aRR, 1.40; 95% CI, 1.19-1.65) and elective cesarean delivery (aRR, 1.39; 95% CI, 1.30-1.49) (Figure). Preeclampsia (aRR, 1.14; 95% CI, 1.01-1.29), induction of labor (aRR, 1.12; 95% CI, 1.06-1.18), emergency cesarean delivery (aRR, 1.16; 95% CI, 1.08-1.25), and postpartum hemorrhage (aRR, 1.13; 95% CI, 1.04-1.22) also showed a statistically significant association with maternal OCD. In BC, only emergency cesarean delivery (aRR, 1.15; 95% CI, 1.01-1.31) and antepartum hemorrhage or placental abruption (aRR, 1.48; 95% CI, 1.03-2.14) showed a statistically significant association.

**Table 2.** Frequencies and Unadjusted Relative Risks of Pregnancy, Delivery, and Neonatal Outcomes Among Mothers With or Without OCD in Sweden (1999-2019) and British Columbia, Canada (2000-2019)

Outcome	Sweden, No./total No. (%)		Unadjusted relative risk (95% CI)	British Columbia, Canada, No./total No. (%)		Unadjusted relative risk (95% CI)
	Pregnancies to mothers without OCD	Pregnancies to mothers with OCD		Pregnancies to mothers without OCD	Pregnancies to mothers with OCD	
**Pregnancy or delivery outcome**						
Gestational diabetes						
Yes	28 119/2 137 348 (1.3)	151/8312 (1.8)	1.39 (1.18-1.63)	75 993/821 759 (9.2)	190/2341 (8.1)	0.88 (0.76-1.01)
No	2 109 229/2 137 348 (98.7)	8161/8312 (98.2)	1 [Reference]	745 766/821 759 (90.8)	2151/2341 (91.9)	1 [Reference]
Preeclampsia						
Yes	58 815/2 137 348 (2.8)	286/8312 (3.4)	1.25 (1.11-1.40)	12 877/821 759 (1.6)	49/2341 (2.1)	1.34 (1.01-1.77)
No	2 078 533/2 137 348 (97.2)	8026/8312 (96.6)	1 [Reference]	808 882/821 759 (98.4)	2292/2341 (97.9)	1 [Reference]
Maternal infection						
Yes	6625/2 137 348 (0.3)	34/8312 (0.4)	1.32 (0.94-1.85)	15 216/821 759 (1.9)	37/2341 (1.6)	0.85 (0.62-1.18)
No	2 130 723/2 137 348 (99.7)	8278/8312 (99.6)	1 [Reference]	806 543/821 759 (98.1)	2304/2341 (98.4)	1 [Reference]
Antepartum hemorrhage or placental abruption						
Yes	9599/2 137 348 (0.4)	41/8312 (0.5)	1.02 (0.80-1.29)	18 467/821 759 (2.2)	69/2341 (2.9)	1.31 (1.04-1.66)
No	2 127 749/2 137 348 (99.6)	8271/8312 (99.5)	1 [Reference]	803 292/821 759 (97.8)	2272/2341 (97.1)	1 [Reference]
Premature rupture of membranes						
Yes	10 903/2 137 348 (0.5)	54/8312 (0.6)	1.27 (0.97-1.66)	38 615/821 759 (4.7)	93/2341 (4.0)	0.85 (0.69-1.04)
No	2 126 445 (99.5)	8258/8312 (99.4)	1 [Reference]	783 144/821 759 (95.3)	2248/2341 (96.0)	1 [Reference]
Induction of labor						
Yes	285 870/2 137 348 (13.4)	1509/8312 (18.2)	1.35 (1.29-1.42)	178 975/821 759 (21.8)	568/2341 (24.3)	1.11 (1.03-1.21)
No	1 838 840/2 137 348 (86.0)	6784/8312 (81.6)	1 [Reference]	642 784/821 759 (78.2)	1773/2341 (75.7)	1 [Reference]
Missing	12 638/2 137 348 (0.6)	19/8312 (0.2)	NA	0	0	NA
Mode of delivery						
Elective cesarean delivery	176 119/2 137 348 (8.2)	1008/8312 (12.1)	1.48 (1.39-1.58)	92 298/821 759 (11.2)	253/2341 (10.8)	1.01 (0.89-1.14)
Emergency cesarean delivery	167 518/2 137 348 (7.8)	784/8312 (9.4)	1.25 (1.16-1.34)	157 002/821 759 (19.1)	524/2341 (22.4)	1.17 (1.07-1.27)
Instrumental delivery	147 324/2 137 348 (6.9)	522/8312 (6.3)	0.97 (0.89-1.06)	83 594/821 759 (10.2)	240/2341 (10.3)	1.05 (0.93-1.19)
Vaginal delivery	1 633 749/2 137 348 (76.4)	5979/8312 (71.9)	1 [Reference]	488 865/821 759 (59.5)	1324/2341 (56.6)	1 [Reference]
Missing	12 638/2 137 348 (0.6)	19/8312 (0.2)	NA	0	0	NA
Postpartum hemorrhage						
Yes	141 938/2 137 348 (6.6)	657/8312 (7.9)	1.19 (1.10-1.29)	64 916/821 759 (7.9)	196/2341 (8.4)	1.06 (0.92-1.22)
No	1 995 410/2 137 348 (93.4)	7655/8312 (92.1)	1 [Reference]	756 843/821 759 (92.1)	2145/2341 (91.6)	1 [Reference]
**Neonatal outcome**						
Perinatal death^a^						
Yes	8281/2 137 348 (0.4)	31/8312 (0.4)	0.96 (0.68-1.37)	3014/819 518 (0.4)	8/2336 (0.3)	0.93 (0.47-1.86)
No	2 129 067/2 137 348 (99.6)	8281/8312 (99.6)	1 [Reference]	818 745/819 518 (99.6)	2333/2336 (99.7)	1 [Reference]
Preterm birth						
Yes	101 633/2 129 579 (4.8)	543/8282 (6.6)	1.37 (1.26-1.49)	64 617/819 518 (7.9)	289/2336 (12.4)	1.57 (1.40-1.76)
No	2 027 946/2 129 579 (95.2)	7739/8282 (93.4)	1 [Reference]	754 901 (92.1)	2047/2336 (87.6)	1 [Reference]
Small for gestational age						
Yes	136 992/2 129 579 (6.4)	588/8282 (7.1)	1.10 (1.02-1.20)	20 558/819 518 (2.5)	58/2336 (2.5)	0.99 (0.76-1.28)
No	1 987 234/2 129 579 (93.3)	7675/8282 (92.7)	1 [Reference]	798 858/819 518 (97.5)	2278/2336 (97.5)	1 [Reference]
Missing	5353/2 129 579 (0.3)	19/8282 (0.2)	NA	102/819 518 (0.01)	0	NA
Low birth weight (<2500 g)						
Yes	65 392/2 129 579 (3.1)	347/8282 (4.2)	1.36 (1.23-1.52)	33 594/819 518 (4.1)	137/2336 (5.9)	1.43 (1.21-1.69)
No	2 060 059/2 129 579 (96.7)	7921/8282 (95.6)	1 [Reference]	785 702/819 518 (95.9)	2199/2336 (94.1)	1 [Reference]
Missing	4128/2 129 579 (0.2)	14/8282 (0.2)	NA	222/819 518 (0.03)	0	NA
Low Apgar score at 5 min						
Yes	36 694/2 129 579 (1.7)	248/8282 (3.0)	1.74 (1.53-1.97)	14 895/819 518 (1.8)	131/2336 (5.6)	3.08 (2.60-3.66)
No	2 092 885/2 129 579 (98.3)	8034/8282 (97.0)	1 [Reference]	802 649/819 518 (97.9)	2200/2336 (94.2)	1 [Reference]
Missing	0	0	NA	1974/819 518 (0.2)	≤5	NA
Neonatal hypoglycemia						
Yes	52 323/2 129 579 (2.5)	271/8282 (3.3)	1.33 (1.18-1.50)	10 967/819 518 (1.3)	48/2336 (2.1)	1.54 (1.16-2.04)
No	2 077 256/2 129 579 (97.5)	8011/8282 (96.7)	1 [Reference]	808 551/819 518 (98.7)	2288/2336 (97.9)	1 [Reference]
Neonatal jaundice						
Yes	98 483/2 129 579 (4.6)	387/8282 (4.7)	1.01 (0.91-1.12)	68 450/819 518 (8.4)	191/2336 (8.2)	0.98 (0.85-1.13)
No	2 031 096/2 129 579 (95.4)	7895/8282 (95.3)	1 [Reference]	751 068/819 518 (91.6)	2145/2336 (91.8)	1 [Reference]
Neonatal respiratory distress						
Yes	70 399/2 129 579 (3.3)	516/8282 (6.2)	1.88 (1.73-2.06)	51 028/819 518 (6.2)	222/2336 (9.5)	1.53 (1.34-1.74)
No	2 059 180/2 129 579 (96.7)	7766/8282 (93.8)	1 [Reference]	768 490/819 518 (93.8)	2114/2336 (90.5)	1 [Reference]
Neonatal infections						
Yes	29 498/2 129 579 (1.4)	136/8282 (1.6)	1.19 (1.00-1.40)	7814/819 518 (1.0)	18/2336 (0.8)	0.81 (0.51-1.28)
No	2 100 081/2 129 579 (98.6)	8146/8282 (98.4)	1 [Reference]	811 704/819 518 (99.0)	2318/2336 (99.2)	1 [Reference]
Congenital malformation						
Major	87 326/2 129 579 (4.1)	410/8282 (5.0)	1.22 (1.11-1.35)	67 784/819 518 (8.3)	287/2336 (12.3)	1.49 (1.33-1.67)
Minor	55 299/2 129 579 (2.6)	304/8282 (3.7)	1.43 (1.27-1.60)	24 896/819 518 (3.0)	77/2336 (3.3)	1.13 (0.91-1.42)
No	1 986 954/2 129 579 (93.3)	7568/8282 (91.4)	1 [Reference]	726 838/819 518 (88.7)	1972/2336 (84.4)	1 [Reference]

Abbreviations: NA, not applicable; OCD, obsessive-compulsive disorder.

^a^ Including stillbirths and neonatal deaths within the first 27 completed days of life. The denominator for perinatal death was all livebirths or stillbirths at 22 completed weeks or later (ie, as in Pregnancy and delivery outcomes above), and the denominator for the remaining variables was live births at 22 completed weeks or later.

**Figure.**
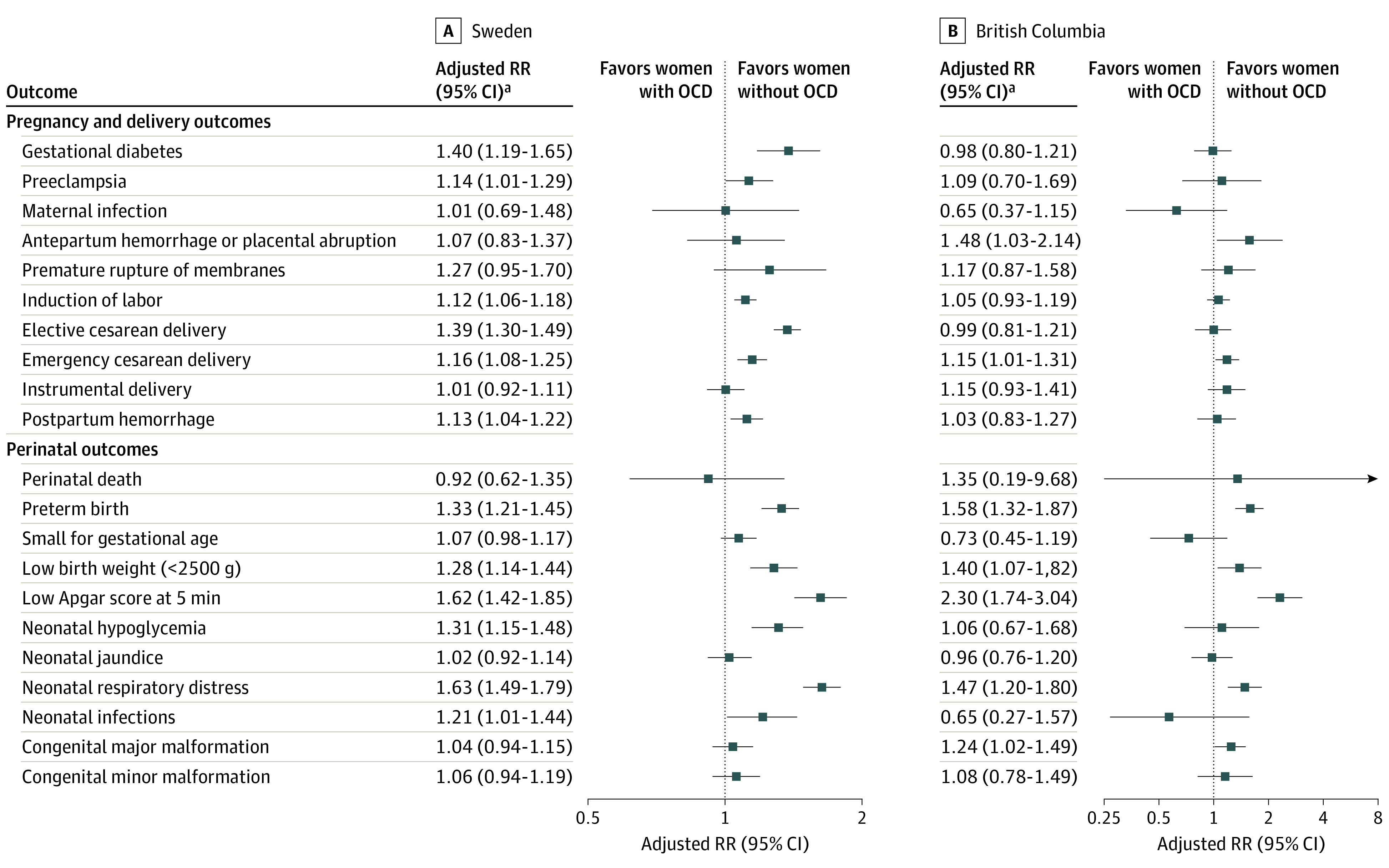
Adjusted Risk Ratios (RRs) of Pregnancy, Delivery, and Neonatal Outcomes Among Mothers With or Without Obsessive-Compulsive Disorder in Sweden (1999-2019) and British Columbia, Canada (2000-2019) ^a^Risk ratios adjusted for age at delivery, parity, place of birth, educational level, cohabitation with a partner, body mass index, prepregnancy diabetes, smoking during pregnancy, and year of delivery.

In Sweden and BC, mothers with OCD had elevated risks of adverse neonatal outcomes compared with women without OCD, including low Apgar score at 5 minutes (Sweden: aRR, 1.62; 95% CI, 1.42-1.85; BC: aRR, 2.30; 95% CI, 1.74-3.04), preterm birth (Sweden: aRR, 1.33; 95% CI, 1.21-1.45; BC: aRR, 1.58; 95% CI, 1.32-1.87), low birth weight (Sweden: aRR, 1.28; 95% CI, 1.14-1.44; BC: aRR, 1.40; 95% CI, 1.07-1.82), and neonatal respiratory distress (Sweden: aRR, 1.63; 95% CI, 1.49-1.79; BC: aRR, 1.47; 95% CI, 1.20-1.80) (Figure). In Sweden, maternal OCD was additionally associated with increased risks of neonatal hypoglycemia (aRR, 1.31; 95% CI, 1.15-1.48) and neonatal infections (aRR, 1.21; 95% CI, 1.01-1.44). The increased risk of major congenital malformations was significant only in BC (aRR, 1.24; 95% CI, 1.02-1.49).

Additional adjustment for psychiatric comorbidity, particularly when accounting for the most common group (ie, mood and anxiety disorders), attenuated the magnitude of the associations in both cohorts, although results remained overall unchanged (eTable 3 in Supplement 1). Furthermore, the association of maternal OCD with pregnancy and neonatal outcomes remained approximately constant over time (eTable 4 in Supplement 1).

### Comparison Between Mothers With OCD Taking SRIs and Mothers Not Taking SRIs

In Sweden, of the 8043 women with OCD with available information on medication (from July 2005 to 2020), 2990 (37.2%) were taking SRIs during pregnancy. In BC, among the 2341 women with OCD with medication information from 2000 to 2020, 1896 (81.0%) were taking SRIs during pregnancy. Compared with Swedish women with OCD not taking SRIs, Swedish women with OCD taking SRIs had increased risks of premature rupture of membranes, emergency cesarean delivery, and postpartum hemorrhage, while in BC women with OCD taking SRIs had increased risks of induction of labor, emergency cesarean delivery, and instrumental delivery, as well as a lower risk of premature rupture of membranes, compared with women with OCD not taking SRIs (Table 3; eTable 5 in Supplement 1). In Sweden, newborns of women with OCD taking SRIs had increased risks of preterm birth, low birth weight, low Apgar score, neonatal hypoglycemia, neonatal respiratory distress, and neonatal infections, compared with those with OCD not taking SRIs. In the BC cohort, only low Apgar score was statistically significant for these comparisons. In supplementary analyses of the Swedish cohort, the comparison of women with OCD who were not taking SRIs during pregnancy (n = 5053) with women without OCD (n = 1 604 483) showed that OCD (without SRIs) was associated with increased risks of induction of labor, elective and emergency cesarean delivery, preterm birth, low birth weight, and neonatal respiratory distress (eTables 5 and 6 in Supplement 1).

**Table 3.** Associations Between Women With OCD Who Were Taking SRIs (vs Those Not Taking SRIs) and Pregnancy, Delivery, and Neonatal Outcomes in Sweden (July 2005-2019) and British Columbia, Canada (2000-2019)

Outcome	Relative risk (95% CI)			
	Sweden		British Columbia, Canada	
	Unadjusted	Adjusted^a^	Unadjusted	Adjusted^a^
**Pregnancy or delivery outcome**				
Gestational diabetes	1.58 (1.14-2.19)	1.33 (0.95-1.85)	1.48 (0.98-2.24)	1.51 (0.92-2.46)
Preeclampsia	1.29 (1.02-1.64)	1.19 (0.93-1.52)	2.64 (0.95-7.34)	Not provided^b^
Maternal infection	1.33 (0.68-2.63)	1.21 (0.57-2.58)	1.21 (0.51-2.91)	1.43 (0.49-4.19)
Antepartum hemorrhage or placental abruption	0.86 (0.52-1.45)	0.90 (0.53-1.53)	0.92 (0.51-1.66)	0.97 (0.46-2.04)
Premature rupture of membranes	1.75 (1.02-3.01)	2.00 (1.12-3.55)	0.47 (0.31-0.72)	0.49 (0.30-0.81)
Induction of labor	0.96 (0.86-1.06)	0.93 (0.83-1.04)	1.26 (1.01-1.58)	1.29 (1.00-1.68)
Elective cesarean delivery	1.15 (1.01-1.30)	1.07 (0.93-1.22)	1.41 (1.00-1.99)	1.31 (0.86-1.98)
Emergency cesarean delivery	1.24 (1.07-1.43)	1.22 (1.05-1.42)	1.32 (1.04-1.66)	1.45 (1.09-1.92)
Instrumental delivery	1.12 (0.93-1.34)	1.14 (0.94-1.37)	1.34 (0.95-1.89)	1.50 (1.00-2.24)
Postpartum hemorrhage	1.32 (1.13-1.54)	1.27 (1.08-1.49)	0.92 (0.65-1.30)	0.86 (0.58-1.27)
**Neonatal outcome**				
Perinatal death	1.48 (0.72-3.03)	1.50 (0.70-3.20)	1.64 (0.20-13.35)	Not provided^b^
Preterm birth	1.43 (1.21-1.70)	1.44 (1.20-1.73)	1.04 (0.78-1.41)	0.95 (0.67-1.35)
Small for gestational age	0.95 (0.80-1.13)	0.97 (0.81-1.16)	1.47 (0.70-3.09)	1.78 (0.61-5.16)
Low birth weight (<2500 g)	1.32 (1.07-1.64)	1.35 (1.08-1.70)	1.05 (0.68-1.62)	1.06 (0.61-1.85)
Low Apgar score at 5 min	2.58 (1.99-3.34)	2.74 (2.07-3.62)	2.13 (1.20-3.78)	2.09 (1.04-4.17)
Neonatal hypoglycemia	1.54 (1.21-1.97)	1.40 (1.09-1.81)	1.64 (0.70-3.86)	1.39 (0.54-3.62)
Neonatal jaundice	1.12 (0.91-1.38)	1.15 (0.92-1.42)	1.26 (0.85-1.86)	1.17 (0.74-1.87)
Neonatal respiratory distress	1.89 (1.58-2.25)	1.89 (1.57-2.26)	1.21 (0.85-1.73)	1.10 (0.72-1.67)
Neonatal infections	1.55 (1.10-2.18)	1.54 (1.07-2.20)	1.17 (0.34-4.05)	Not provided^b^
Major congenital malformation	0.96 (0.78-1.18)	0.98 (0.79-1.22)	1.10 (0.81-1.49)	1.06 (0.75-1.51)
Minor congenital malformation	0.96 (0.75-1.21)	0.96 (0.75-1.24)	1.59 (0.82-3.09)	1.41 (0.69-2.89)

Abbreviations: OCD, obsessive-compulsive disorder; SRI, serotonin reuptake inhibitor.

^a^ Adjusted for age at delivery, parity, place of birth, educational level, cohabitation with a partner, body mass index, prepregnancy diabetes (only in the Swedish cohort, due to low numbers in the British Columbia cohort), smoking during pregnancy, and year of delivery.

^b^ Only unadjusted relative risks are provided (due to limited statistical power).

### Sister and Cousin Analyses

The Swedish subcohort used to compare pregnancy, delivery, and neonatal outcomes for women with OCD (n = 2710) vs their full sisters without OCD (n = 4780) revealed weaker associations than the population-based analysis, based on examining the point estimates between the population cohort and the sister cohort (Table 4). Only the associations between maternal OCD and emergency cesarean delivery (aOR, 1.30; 95% CI, 0.99-1.72), low Apgar score (aOR, 1.89; 95% CI, 1.27-2.80), and neonatal respiratory distress (aOR, 1.74; 95% CI, 1.29-2.35) remained statistically significant. However, other associations were similar (eg, induction of labor, postpartum hemorrhage) or even larger in magnitude (eg, gestational diabetes, neonatal infections) than those in the primary analyses, although they had wide 95% CIs and were not significant, likely due to the smaller number of participants.

**Table 4.** Pregnancy, Delivery, and Neonatal Outcomes Among Mothers With OCD and Their Offspring and Their Full Sisters Without OCD and Their Offspring (Cousins) in Sweden (1999-2019)

Outcome	No./total No. (%)		Odds ratio (95% CI)	
	Pregnancies to mothers with OCD	Pregnancies to unexposed sisters of mothers with OCD	Unadjusted	Adjusted^a^
**Pregnancy or delivery outcome**				
Gestational diabetes				
Yes	41/2710 (1.5)	40/4780 (0.8)	2.27 (1.38-3.73)	1.66 (0.86-3.23)
No	2669/2710 (98.5)	4740/4780 (99.2)	1 [Reference]	1 [Reference]
Preeclampsia				
Yes	89/2710 (3.3)	150/4780 (3.1)	1.06 (0.79-1.42)	0.98 (0.67-1.44)
No	2621/2710 (96.7)	4630/4780 (96.9)	1 [Reference]	1 [Reference]
Maternal infection				
Yes	6/2710 (0.2)	13/4780 (0.3)	0.84 (0.30-2.36)	1.26 (0.23-6.99)
No	2704/2710 (99.8)	4767/4780 (99.7)	1 [Reference]	1 [Reference]
Antepartum hemorrhage or placental abruption				
Yes	11/2710 (0.4)	18/4780 (0.4)	0.80 (0.42-1.52)	0.74 (0.32-1.71)
No	2699/2710 (99.6)	4762/4780 (99.6)	1 [Reference]	1 [Reference]
Premature rupture of membranes				
Yes	18/2710 (0.7)	28/4780 (0.6)	1.24 (0.66-2.33)	0.97 (0.40-2.38)
No	2692/2710 (99.3)	4752/4780 (99.4)	1 [Reference]	1 [Reference]
Induction of labor				
Yes	484/2710 (17.9)	635/4780 (13.3)	1.54 (1.33-1.78)	1.15 (0.97-1.37)
No	2218/2710 (81.8)	4119/4780 (86.2)	1 [Reference]	1 [Reference]
Missing	8/2710 (0.3)	26/4780 (0.5)	NA	NA
Mode of delivery				
Elective cesarean delivery	300/2710 (11.1)	400/4780 (8.4)	1.46 (1.20-1.79)	1.18 (0.93-1.50)
Emergency cesarean delivery	237/2710 (8.7)	321/4780 (6.7)	1.45 (1.16-1.80)	1.30 (0.99-1.72)
Instrumental delivery	171/2710 (6.3)	344/4780 (7.2)	0.83 (0.66-1.04)	0.96 (0.72-1.29)
Vaginal delivery	1994/2710 (73.6)	3689/4780 (77.2)	1 [Reference]	1 [Reference]
Missing	8/2710 (0.3)	26/4780 (0.5)	NA	NA
Postpartum hemorrhage				
Yes	206/2710 (7.6)	305/4780 (6.4)	1.23 (1.01-1.50)	1.15 (0.91-1.45)
No	2504/2710 (92.4)	4475/4780 (93.6)	1 [Reference]	1 [Reference]
**Neonatal outcome**				
Perinatal death^b^				
Yes	9/2710 (0.3)	15/44780 (0.3)	1.40 (0.58-3.38)	1.20 (0.40-3.61)
No	2701/2710 (99.7)	4765/4780 (99.7)	1 [Reference]	1 [Reference]
Preterm birth				
Yes	160/2702 (5.9)	244/4766 (5.1)	1.20 (0.96-1.50)	1.16 (0.88-1.53)
No	2542/2702 (94.1)	4522/4766 (94.9)	1 [Reference]	1 [Reference]
Small for gestational age				
Yes	196/2702 (7.3)	299/4766 (6.3)	1.18 (0.96-1.45)	1.14 (0.88-1.47)
No	2499/2702 (92.5)	4456/4766 (93.5)	1 [Reference]	1 [Reference]
Missing	7/2702 (0.3)	11/4766 (0.2)	NA	NA
Low birth weight (<2500 g)				
Yes	112/2702 (4.1)	174/4766 (3.7)	1.09 (0.84-1.42)	0.92 (0.65-1.31)
No	2586/2702 (95.7)	4583/4766 (96.2)	1 [Reference]	1 [Reference]
Missing	≤5	9/4766 (0.2)	NA	NA
Low Apgar score at 5 min				
Yes	91/2702 (3.4)	84/4766 (1.8)	1.75 (1.27-2.42)	1.89 (1.27-2.80)
No	2611/2702 (96.6)	4682/4766 (98.2)	1 [Reference]	1 [Reference]
Neonatal hypoglycemia				
Yes	71/2702 (2.6)	142/4766 (3.0)	0.81 (0.59-1.11)	0.86 (0.59-1.24)
No	2631/2702 (97.4)	4624/4766 (97.0)	1 [Reference]	1 [Reference]
Neonatal jaundice				
Yes	117/2702 (4.3)	227/4766 (4.8)	0.96 (0.75-1.23)	0.96 (0.71-1.32)
No	2585/2702 (95.7)	4539/4766 (95.2)	1 [Reference]	1 [Reference]
Neonatal respiratory distress				
Yes	141/2702 (5.2)	161/4766 (3.4)	1.59 (1.23-2.04)	1.74 (1.29-2.35)
No	2561/2702 (94.8)	4605/4766 (96.6)	1 [Reference]	1 [Reference]
Neonatal infections				
Yes	42/2702 (1.6)	53/4766 (1.1)	1.30 (0.84-2.00)	1.48 (0.81-2.72)
No	2660/2702 (98.4)	4713/4766 (98.9)	1 [Reference]	1 [Reference]
Congenital malformation				
Major	111/2702 (4.1)	219/4766 (4.6)	0.84 (0.66-1.08)	0.88 (0.66-1.17)
No	2591/2702 (95.9)	4547/4766 (95.4)	1 [Reference]	1 [Reference]

Abbreviations: NA, not applicable; OCD, obsessive-compulsive disorder.

^a^ Adjusted for age at delivery, parity, place of birth, educational level, cohabitation with a partner, body mass index, prepregnancy diabetes, smoking during pregnancy, and year of delivery.

^b^ Including stillbirths and neonatal deaths within the first 27 completed days of life. The denominator for perinatal death was all livebirth or stillbirth at 22 completed weeks or later (ie, as in Pregnancy and delivery outcomes above) and the denominator for the remaining variables was live births at 22 completed weeks or later.

## Discussion

In this population-based study in Sweden and BC, Canada, women with OCD had higher risks of gestational diabetes, preeclampsia, antepartum hemorrhage or placental abruption, induction of labor, elective and emergency cesarean delivery, and postpartum hemorrhage, and their newborns had higher risks of preterm birth, low birth weight, low 5-minute Apgar score, neonatal hypoglycemia, neonatal respiratory distress, neonatal infections, and major congenital malformations. Women with OCD had higher rates of psychiatric comorbidity; controlling for these comorbidities attenuated but did not eliminate the increased risks. Sister and cousin analyses suggested that at least some of the associations identified in the primary analyses were not attributable to familial confounding.

In line with our results, higher risks of preeclampsia,^8^ cesarean delivery,^8^ preterm birth,^7,8^ and low birth weight^4,5^ have previously been reported in the small body of literature focusing on maternal OCD and perinatal and neonatal outcomes.^4,5,6,7,8^ We observed a differential frequency in the dispensation of SRIs during pregnancy for women with OCD across cohorts (37.2% in Sweden vs 81.0% in BC). Despite this difference, women with OCD who were dispensed SRIs during pregnancy showed an increased risk of several pregnancy, delivery, and neonatal outcomes in both cohorts, compared with women with OCD not taking SRIs during pregnancy. This finding requires cautious interpretation due to potential confounding by indication.^31,32^ Women with OCD who are prescribed SRIs may represent a group with a more severe form of the disorder. A recent review found evidence of associations between SSRI use during pregnancy and increased risks of preterm birth, neonatal respiratory distress, and cardiovascular malformations.^11^ Nevertheless, the reviewed studies represent evidence of modest quality as they use nonexperimental designs, which cannot adequately control for confounding by indication. In our study, the subcohort of women with OCD who were not taking SRIs during pregnancy still presented increased risks of adverse perinatal and neonatal outcomes, suggesting that our observations are not entirely explained by medication use during pregnancy. With the current evidence, women with OCD should not be advised to discontinue pharmacologic treatment when it is clinically indicated.^10^ More research is needed to ascertain the association of different types and doses of SRI drugs during pregnancy with outcomes in this patient group.

Most of the associations in sister- and cousin-controlled analyses did not reach statistical significance, likely due to the smaller number of participants in these analyses. Regardless, for some of the outcomes, familial confounding seemed to explain the associations found in the main analysis (eg, preeclampsia, neonatal hypoglycemia). However, several other outcomes showed robust associations with maternal OCD (eg, low Apgar score, neonatal respiratory distress) or associations that, despite not being statistically significant, were similar (eg, induction of labor, postpartum hemorrhage) or even stronger (eg, gestational diabetes, neonatal infections), based on observing the point estimates, than those in the primary analyses. This finding implies that the increased risk of these outcomes in women with OCD is at least partially independent from shared genes or environment. Some of the associations of maternal OCD with pregnancy and neonatal outcomes might be through direct intrauterine mechanisms, such as medication use during pregnancy. In turn, some of the perinatal outcomes associated with maternal OCD (eg, cesarean delivery, low Apgar score) have shown to be risk factors for OCD later in life.^33^ Further studies of familial coaggregation that can disentangle potential etiologic factors are warranted.

Our findings have public health and clinical implications. There is a need for improved collaboration and a better collaboration between psychiatry and obstetric services for the management of care for women with OCD. Given the described associations between maternal OCD and adverse maternal and neonatal outcomes, particularly in women receiving SRIs, close surveillance of pregnant women with OCD as well as monitored deliveries and special attention to neonatal care in their offspring are warranted.

### Strengths and Limitations

This study has some strengths. It is, to our knowledge, the largest assessment to date of perinatal and neonatal outcomes for women with OCD and their offspring. We used cohorts from 2 different countries with data spanning over 20 years, and our large primary analyses were supplemented by a sister and cousin design, which allowed us to examine potential familial confounding.

Our study also has a few limitations. First, our exposed cohorts included only women who sought medical care for OCD. Moreover, in Sweden, only diagnoses made in specialist care were available, with information from outpatient care from 2001 onward, which resulted in an increased number of women with OCD over the study period. However, the associations remained approximately constant over time. Second, the frequency of some of the outcomes was relatively low, particularly for analyses conducted in subcohorts (eg, SRI use and sister and cousin analyses). Third, the drug registers provided information on the dispensation of medication, but we were not able to ascertain drug adherence. Furthermore, we did not examine the association of other medication groups (eg, antipsychotics and benzodiazepines) with outcomes, although we did adjust for psychiatric comorbidity. Fourth, the BC cohort was significantly smaller than the Swedish cohort, and power issues, together with different obstetric practices between countries, may be responsible for some of the dissimilar findings. Fifth, the family analyses could be confounded by unmeasured factors that make siblings and cousins different from one another.^34^

## Conclusions

In these cohort studies, maternal OCD was associated with an increased risk of pregnancy, delivery, and, most notably, neonatal outcomes. Women with OCD taking SRIs during pregnancy appeared to have higher risks of these outcomes, but medication alone was unlikely to completely explain the findings. Several of the observed associations were at least partially independent from familial confounding. Improved collaboration between psychiatry and obstetric services, as well as improved maternal and neonatal care for women with OCD and their children, is warranted.

## Notes

Supplement 1. **eTable 1.** *International Statistical Classification of Diseases and Related Health Problems*, 9^th^ and 10^th^ edition Codes Used to Identify Study Exposure, Outcomes, and Covariates in the Study **eTable 2.** Definition of Outcome Variables and Covariates in the Study **eTable 3.** Associations Between Maternal Obsessive-Compulsive Disorder (OCD) and Pregnancy, Delivery, and Neonatal Outcomes in Sweden (From 1999 to 2019) and in British Columbia, Canada (From 2000 to 2019), Additionally Adjusting for Psychiatric Comorbidity **eTable 4.** Associations Between Maternal Obsessive-Compulsive Disorder (OCD) and Pregnancy, Delivery, and Neonatal Outcomes in Sweden (From 1999 to 2019), by Year of Birth **eTable 5.** Frequencies of Study Outcomes According to Obsessive-Compulsive Disorder (OCD) Status (Sweden Only) and Dispensation of SRIs During Pregnancy in Mothers With OCD in Sweden (From July 2005 to 2019) and in British Columbia, Canada (From 2000 to 2019) **eTable 6.** Pregnancy, Delivery, and Neonatal Outcomes in Pregnancies and Births to Mothers With Obsessive-Compulsive Disorder (OCD) That Were Not on Serotonin Reuptake Inhibitors During Pregnancy in Sweden (From July 2005 to 2019), Compared With Mothers Who Did Not Have OCD

Supplement 2.
Data Sharing Statement
