# Supplementary material for: Pregnancy, Delivery, and Neonatal Outcomes Associated With Maternal Obsessive-Compulsive Disorder: Two Cohort Studies in Sweden and British Columbia, Canada
Source: JAMA Netw Open. 2023 Jun 14;6(6):e2318212. doi: 10.1001/jamanetworkopen.2023.18212 (PMC10267772; doi:10.1001/jamanetworkopen.2023.18212)
Supplement: Supplement 1. — eTable 1. International Statistical Classification of Diseases and Related Health Problems, 9th and 10th edition Codes Used to Identify Study Exposure, Outcomes, and Covariates in the Study eTable 2. Definition of Outcome Variables and Covariates in the Study eTable 3. Associations Between Maternal Obsessive-Compulsive Disorder (OCD) and Pregnancy, Delivery, and Neonatal Outcomes in Sweden (From 1999 to 2019) and in British Columbia, Canada (From 2000 to 2019), Additionally Adjusting for Psychiatric Comorbidity eTable 4. Associations Between Maternal Obsessive-Compulsive Disorder (OCD) and Pregnancy, Delivery, and Neonatal Outcomes in Sweden (From 1999 to 2019), by Year of Birth eTable 5. Frequencies of Study Outcomes According to Obsessive-Compulsive Disorder (OCD) Status (Sweden Only) and Dispensation of SRIs During Pregnancy in Mothers With OCD in Sweden (From July 2005 to 2019) and in British Columbia, Canada (From 2000 to 2019) eTable 6. Pregnancy, Delivery, and Neonatal Outcomes in Pregnancies and Births to Mothers With Obsessive-Compulsive Disorder (OCD) That Were Not on Serotonin Reuptake Inhibitors During Pregnancy in Sweden (From July 2005 to 2019), Compared With Mothers Who Did Not Have OCD [file jamanetwopen-e2318212-s001.pdf]

Supplementary Online Content

Fernández de la Cruz L, Joseph KS, Wen Q, Stephansson O, Mataix-Cols D, Razaz N. Pregnancy, delivery, and neonatal outcomes associated with maternal obsessive-compulsive disorder: two cohort studies in Sweden and British Columbia, Canada. *JAMA Netw Open.* 2023;6(6):e2318212. doi:10.1001/jamanetworkopen.2023.18212

eTable 1. *International Statistical Classification of Diseases and Related Health Problems*, 9th and 10th edition Codes Used to Identify Study Exposure, Outcomes, and Covariates in the Study
eTable 2. Definition of Outcome Variables and Covariates in the Study
eTable 3. Associations Between Maternal Obsessive-Compulsive Disorder (OCD) and Pregnancy, Delivery, and Neonatal Outcomes in Sweden (From 1999 to 2019) and in British Columbia, Canada (From 2000 to 2019), Additionally Adjusting for Psychiatric Comorbidity
eTable 4. Associations Between Maternal Obsessive-Compulsive Disorder (OCD) and Pregnancy, Delivery, and Neonatal Outcomes in Sweden (From 1999 to 2019), by Year of Birth
eTable 5. Frequencies of Study Outcomes According to Obsessive-Compulsive Disorder (OCD) Status (Sweden Only) and Dispensation of SRIs During Pregnancy in Mothers With OCD in Sweden (From July 2005 to 2019) and in British Columbia, Canada (From 2000 to 2019)
eTable 6. Pregnancy, Delivery, and Neonatal Outcomes in Pregnancies and Births to Mothers With Obsessive-Compulsive Disorder (OCD) That Were Not on Serotonin Reuptake Inhibitors During Pregnancy in Sweden (From July 2005 to 2019), Compared With Mothers Who Did Not Have OCD

This supplementary material has been provided by the authors to give readers additional information about their work.

© 2023 Fernández de la Cruz et al. *JAMA Network Open.*

eTable 1. *International Statistical Classification of Diseases and Related Health Problems,* 9th and 10th edition Codes Used to Identify Study Exposure, Outcomes, and Covariates in the Study

**Exposure**	**ICD codes**
Obsessive-compulsive disorder[a,b]	ICD-9: 300.3 (300D); ICD-10: F42
**Outcomes**	**ICD codes**[c]
Pregnancy and delivery outcomes[d]	
Gestational diabetes	ICD-9: 648.8; ICD-10: O24.4
Preeclampsia	ICD-9: 642.4, 642.5, 642.6; ICD-10: O14-O15
Maternal infection	ICD-9: 659.3, 658.4; ICD-10: O41.1, O75.3
Antepartum hemorrhage	ICD-9: 641.2, 641.3, 641.8, 641.9; ICD-10: O44, O45, O46
Premature rupture of membranes	ICD-9: 658.1; ICD-10: O42.1
Postpartum hemorrhage	ICD-9: 666; ICD-10: O67.0, O67.8, O72
Neonatal outcomes[a,e]	
Neonatal hypoglycemia	ICD-9: 775.6; ICD-10: P70.4
Neonatal jaundice	ICD-9: 773, 774; ICD-10: P55-P59
Neonatal respiratory distress	ICD-9: 769, 770.6, 770.8; ICD-10: P22
Neonatal infections	ICD-9: 771; ICD-10: P35-P39
Congenital malformation:	
Minor malformations[f]	ICD-9: 7441, 7444, 7469, 7470, 7475, 7500, 7501, 7525, 7543, 7546, 7556, 7550, 7551, 7561, 7569, 7573, 7579; ICD-10: Q17.0, Q17.5, Q18.0, Q18.1, Q25.0, Q27.0, Q31.5, Q32.0, Q52.3, Q53.0 to Q53.9, Q66.5 to Q66.9, Q69.0 to Q69.9, Q70.0 to Q70.9, Q76.0, Q79.9, Q82.5, and Q82.9
Major malformations	ICD-9: 74, 75; ICD-10: Q00-Q99, except for the codes above for minor malformations
**Covariates**	**ICD codes**[c]
Pre-pregnancy hypertension[g]	ICD-9: 401 to 405, 642.2, 642.7; ICD-10: I10 to I15, O10 and O11
Pre-pregnancy diabetes	ICD-9: 250, 648.0; ICD-10: E10-14, O24.1-24.3
Psychiatric comorbidities:[h]	
Manic episode or bipolar disorder + schizophrenia or other psychotic disorders[b,i]	ICD-9: 296.0 (296A), 296.2 (296C), 296.3 (296D), 296.4 (296E), 296.5 (296W), 296.6 (296X); ICD-10: F25.0, F30, F31, F34.0
	ICD-9: 295, minus 295.5 (295F), 297, 298, minus 298.0 (298A); ICD-10: F20, F21, F22, F23, F24, F25, minus F25.0, F28, F29
Depression or other mood disorder + phobic, anxiety, reaction to severe stress or adjustment disorders[b,j]	ICD-9: 296.1 (296B), 298.0 (298A), 300.4 (300E), 311; ICD-10: F32, F33, F34, minus F34.0, F38, F39
	ICD-9: 300.0 (300A), 300.2 (300C), 308, 309; ICD-10: F40, F41, F43

[a]Identified from the National Patient Register (Swedish cohort) or the Perinatal Database (British Columbia cohort). [b]In a parenthesis, the ICD-9 code for the Swedish version of the ICD, if differing from the International ICD version. [c]ICD-9 codes used to identify all outcomes, pre-pregnancy hypertension, and pre-pregnancy

© 2023 Fernández de la Cruz et al. *JAMA Network Open.*

diabetes only in the British Columbia, Canada cohort during the year 2000. [d]Identified from the Medical Birth Register and the National Patient Register (Sweden) or the Perinatal Database and the Medical services plan or the Hospital Discharge Data (British Columbia, Canada), as appropriate. [e]Information on neonatal outcomes, besides malformations, was restricted to the first 27 days of life. [f]Minor malformations as defined by the Swedish National Board of Health and Welfare. [g]Definition of pre-pregnancy hypertension also included self-reported information at the first antenatal visit. [h]For consistency with the Swedish outpatient register, in BC, psychiatric disorders in the Medical Services Plans were only identified if the diagnoses had a specialist code such as "Psychiatry", "Psychotherapy" or "Neuropsychiatry". [i]Comorbidities in this group were considered if recorded between age 10 or older, to avoid potential misclassifications, and the index childbirth. [j]Comorbidities in this group were considered if recorded between age 6 or older, to avoid potential misclassifications, and the index childbirth.

© 2023 Fernández de la Cruz et al. *JAMA Network Open.*

eTable 2. Definition of Outcome Variables and Covariates in the Study

Outcomes	Definition
Pregnancy and delivery outcomes	
Mode of delivery	Classified as vaginal delivery, instrumental vaginal delivery (use of forceps or vacuum extraction), elective cesarean section (if performed before labor onset) or emergency cesarean section.
Neonatal outcomes	
Perinatal death	Includes still births and neonatal deaths within the first 27 completed days of life.
Preterm birth	Categorized as birth earlier than 37 completed weeks' gestation.
Small for gestational age	Below the 10th percentile as defined using the current Swedish or Canadian standard for normal fetal growth.
Low birth weight	Defined as <2500 gr.
Low Apgar score at 5 minutes	Score of 4-6.
**Covariates**	**Definitions**
Education level	Highest level of education achieved, extracted from the National Education Register (Swedish cohort only).
Cohabitation with a partner	Self-reported at the first antenatal visit.
Parity	Defined as the number of births to each mother.
Smoking during pregnancy	Mothers who reported daily smoking at the first antenatal visit and/or at 30 to 32 gestational weeks were classified as smokers. The rest were classified as non-smokers.

© 2023 Fernández de la Cruz et al. *JAMA Network Open.*

eTable 3. Associations Between Maternal Obsessive-Compulsive Disorder (OCD) and Pregnancy, Delivery, and Neonatal Outcomes in Sweden (from 1999 to 2019) and in British Columbia, Canada (from 2000 to 2019), Additionally Adjusting for Psychiatric Comorbidity

	Sweden				British Columbia, Canada			
**Pregnancy and delivery outcomes**	**Adjusted RR (95% CI)[a]**	**RR additionally adjusted for bipolar and psychotic disorders (95% CI)[b]**	**RR additionally adjusted for mood and anxiety disorders (95% CI)[c]**	**RR additionally adjusted for any psychiatric comorbidity (95% CI)[d]**	**Adjusted RR (95% CI)[a]**	**RR additionally adjusted for bipolar and psychotic disorders (95% CI)[b]**	**RR additionally adjusted for mood and anxiety disorders (95% CI)[c]**	**RR additionally adjusted for any comorbidity (95% CI)[d]**
Gestational diabetes	1.40 (1.19-1.65)	1.38 (1.17-1.62)	1.19 (1.01-1.40)	1.19 (1.01-1.41)	0.98 (0.80-1.21)	0.96 (0.78-1.19)	0.94 (0.76-1.16)	0.94 (0.76-1.16)
Preeclampsia	1.14 (1.01-1.29)	1.15 (1.02-1.30)	1.10 (0.97-1.25)	1.11 (0.98-1.25)	1.09 (0.70-1.69)	1.03 (0.66-1.60)	0.98 (0.63-1.53)	0.98 (0.63-1.52)
Maternal infection	1.01 (0.69-1.48)	0.97 (0.66-1.43)	0.89 (0.60-1.32)	0.89 (0.60-1.31)	0.65 (0.37-1.15)	0.65 (0.37-1.15)	0.63 (0.36-1.12)	0.63 (0.36-1.12)
Antepartum hemorrhage / placental abruption	1.07 (0.83-1.37)	1.08 (0.84-1.38)	0.97 (0.75-1.25)	0.97 (0.76-1.25)	1.48 (1.03-2.14)	1.46 (1.01-2.10)	1.37 (0.95-1.98)	1.36 (0.94-1.96)
Premature rupture of membranes	1.27 (0.95-1.70)	1.25 (0.93-1.68)	1.18 (0.88-1.59)	1.17 (0.87-1.58)	1.17 (0.87-1.58)	1.15 (0.86-1.55)	1.12 (0.84-1.51)	1.12 (0.83-1.51)
Induction of labor	1.12 (1.06-1.18)	1.09 (1.03-1.15)	0.99 (0.94-1.04)	0.99 (0.94-1.04)	1.05 (0.93-1.19)	1.04 (0.92-1.18)	1.03 (0.91-1.17)	1.03 (0.91-1.17)
Elective cesarean section	1.39 (1.30-1.49)	1.35 (1.27-1.44)	1.13 (1.06-1.21)	1.13 (1.06-1.21)	0.99 (0.81-1.21)	0.98 (0.80-1.20)	0.97 (0.79-1.19)	0.97 (0.79-1.19)
Emergency cesarean section	1.16 (1.08-1.25)	1.15 (1.06-1.23)	1.06 (0.98-1.14)	1.06 (0.98-1.14)	1.15 (1.01-1.31)	1.13 (0.99-1.29)	1.11 (0.97-1.26)	1.11 (0.97-1.26)
Instrumental delivery	1.01 (0.92-1.11)	1.01 (0.93-1.11)	1.01 (0.92-1.11)	1.01 (0.92-1.11)	1.15 (0.93-1.41)	1.13 (0.92-1.40)	1.13 (0.92-1.40)	1.13 (0.92-1.40)
Postpartum hemorrhage	1.13 (1.04-1.22)	1.12 (1.03-1.21)	1.07 (0.99-1.16)	1.07 (0.99-1.16)	1.03 (0.83-1.27)	1.04 (0.84-1.28)	1.02 (0.82-1.26)	1.02 (0.82-1.26)
	**Sweden**				**British Columbia, Canada**			
**Neonatal outcomes**	**Adjusted RR (95% CI)[a]**	**RR additionally adjusted for bipolar and psychotic disorders (95% CI)[b]**	**RR additionally adjusted for mood and anxiety disorders (95% CI)[c]**	**RR additionally adjusted for any psychiatric comorbidity (95% CI)[d]**	**Adjusted RR (95% CI)[a]**	**RR additionally adjusted for bipolar and psychotic disorders (95% CI)[b]**	**RR additionally adjusted for mood and anxiety disorders (95% CI)[c]**	**RR additionally adjusted for any comorbidity (95% CI)[d]**
Perinatal death[e]	0.92 (0.62-1.35)	0.91 (0.62-1.35)	0.95 (0.64-1.40)	0.94 (0.64-1.39)	1.35 (0.19-9.68)	1.31 (0.18-9.45)	1.17 (0.16-8.47)	1.20 (0.17-8.64)
Preterm birth	1.33 (1.21-1.45)	1.28 (1.17-1.41)	1.11 (1.02-1.22)	1.11 (1.02-1.22)	1.58 (1.32-1.87)	1.51 (1.27-1.80)	1.40 (1.17-1.66)	1.39 (1.17-1.66)
Small for gestational age	1.07 (0.98-1.17)	1.08 (0.99-1.17)	1.08 (0.99-1.18)	1.08 (0.99-1.18)	0.73 (0.45-1.19)	0.72 (0.44-1.17)	0.73 (0.45-1.20)	0.72 (0.44-1.18)
Low birth weight (<2500 g)	1.28 (1.14-1.44)	1.26 (1.12-1.41)	1.14 (1.01-1.28)	1.14 (1.01-1.28)	1.40 (1.07-1.82)	1.35 (1.03-1.76)	1.27 (0.97-1.66)	1.27 (0.97-1.66)
Low Apgar score at 5 minutes	1.62 (1.42-1.85)	1.55 (1.35-1.77)	1.42 (1.24-1.63)	1.42 (1.24-1.63)	2.30 (1.74-3.04)	2.16 (1.63-2.86)	1.96 (1.48-2.60)	1.95 (1.47-2.58)
Neonatal hypoglycemia	1.31 (1.15-1.48)	1.28 (1.13-1.45)	1.16 (1.02-1.31)	1.15 (1.01-1.31)	1.06 (0.67-1.68)	1.04 (0.65-1.65)	0.94 (0.59-1.50)	0.94 (0.59-1.50)
Neonatal jaundice	1.02 (0.92-1.14)	1.01 (0.90-1.12)	0.93 (0.84-1.04)	0.93 (0.84-1.04)	0.96 (0.76-1.20)	0.94 (0.75-1.18)	0.90 (0.72-1.13)	0.90 (0.72-1.13)
Neonatal respiratory distress	1.63 (1.49-1.79)	1.54 (1.41-1.69)	1.34 (1.22-1.47)	1.33 (1.21-1.46)	1.47 (1.20-1.80)	1.40 (1.14-1.72)	1.32 (1.08-1.62)	1.31 (1.07-1.61)
Neonatal infections	1.21 (1.01-1.44)	1.17 (0.98-1.40)	1.15 (0.96-1.38)	1.15 (0.96-1.38)	0.65 (0.27-1.57)	0.59 (0.24-1.42)	0.59 (0.25-1.43)	0.58 (0.24-1.40)

© 2023 Fernández de la Cruz et al. *JAMA Network Open.*

Major congenital malformation	1.04 (0.94-1.15)	1.02 (0.92-1.14)	0.97 (0.87-1.08)	0.97 (0.87-1.07)	1.24 (1.02-1.49)	1.20 (0.99-1.45)	1.17 (0.97-1.42)	1.16 (0.96-1.41)
Minor congenital malformation	1.06 (0.94-1.19)	1.05 (0.93-1.19)	1.01 (0.90-1.14)	1.01 (0.90-1.14)	1.08 (0.78-1.49)	1.07 (0.77-1.48)	1.08 (0.78-1.49)	1.08 (0.78-1.50)

[a]Adjusted model included adjustment for age at delivery, parity, place of birth, education level, cohabitation with a partner, BMI, pre-pregnancy diabetes, smoking during pregnancy, and year of delivery, as reported in Figure 1 in the main manuscript. [b]Adjusted for the variables in the first adjusted model and additionally for bipolar and psychotic disorders (i.e., a record of a manic episode or bipolar disorder, or a record of schizophrenia or other psychotic disorders). [c]Adjusted for the variables in the first adjusted model and additionally for mood and anxiety disorders (i.e., a record of depression or other mood disorders, or a record of phobic, anxiety, reaction to severe stress or adjustment disorders; see ICD codes in eTable 1). [d]Adjusted for the variables in the first adjusted model and additionally for any maternal psychiatric comorbidity, including bipolar and psychotic disorders and/or mood and anxiety disorders. [e]Including stillbirths and neonatal deaths within the first 27 completed days after birth.
Abbreviations: RR, relative risk.

© 2023 Fernández de la Cruz et al. *JAMA Network Open.*

eTable 4. Associations Between Maternal Obsessive-Compulsive Disorder (OCD) and Pregnancy, Delivery, and Neonatal Outcomes in Sweden (from 1999 to 2019), by Year of Birth

	Year of Birth							
	1999-2003 (n = 437,972)		2004-2008 (n = 497,437)		2009-2013 (n = 536,238)		2014-2019 (n = 666,722)	
**Pregnancy and delivery outcomes**	**Unadjusted relative risk (95% CI)**	**Adjusted[a] relative risk (95% CI)**	**Unadjusted relative risk (95% CI)**	**Adjusted[a] relative risk (95% CI)**	**Unadjusted relative risk (95% CI)**	**Adjusted[a] relative risk (95% CI)**	**Unadjusted relative risk (95% CI)**	**Adjusted[a] relative risk (95% CI)**
Gestational diabetes	1.86 (0.60-5.77)	2.50 (0.81-7.77)	1.75 (1.06-2.91)	1.79 (1.06-3.03)	0.99 (0.68-1.45)	1.17 (0.80-1.72)	1.12 (0.92-1.35)	1.38 (1.13-1.67)
Preeclampsia	1.02 (0.42-2.44)	0.90 (0.34-2.40)	1.06 (0.71-1.58)	1.02 (0.67-1.56)	1.29 (1.04-1.59)	1.14 (0.92-1.43)	1.30 (1.12-1.51)	1.16 (0.99-1.36)
Maternal infection	0	0	0.43 (0.06-3.04)	0.45 (0.06-3.21)	1.60 (0.86-2.98)	1.36 (0.68-2.74)	1.18 (0.78-1.78)	0.98 (0.61-1.57)
Antepartum hemorrhage / placental abruption	1.36 (0.34-5.45)	0.79 (0.11-5.63)	1.27 (0.66-2.44)	1.24 (0.62-2.48)	0.85 (0.52-1.38)	0.93 (0.57-1.52)	1.11 (0.81-1.51)	1.12 (0.82-1.55)
Premature rupture of membranes	0.97 (0.14-6.86)	≤5	0.83 (0.31-2.21)	0.67 (0.22-2.07)	2.13 (1.46-3.11)	1.92 (1.29-2.88)	1.17 (0.77-1.78)	1.02 (0.64-1.62)
Induction of labor	1.51 (1.02-2.24)	1.28 (0.82-2.01)	1.09 (0.90-1.33)	1.04 (0.85-1.28)	1.13 (1.02-1.25)	1.10 (0.99-1.22)	1.20 (1.13-1.28)	1.12 (1.05-1.20)
Elective cesarean section	1.15 (0.67-1.98)	1.08 (0.58-2.01)	1.50 (1.24-1.81)	1.46 (1.19-1.79)	1.40 (1.24-1.58)	1.38 (1.21-1.56)	1.43 (1.32-1.54)	1.41 (1.30-1.53)
Emergency cesarean section	1.19 (0.70-2.01)	1.15 (0.65-2.02)	1.10 (0.87-1.38)	1.02 (0.79-1.30)	1.18 (1.03-1.35)	1.12 (0.98-1.29)	1.30 (1.19-1.42)	1.21 (1.10-1.34)
Instrumental delivery	1.23 (0.74-2.04)	0.89 (0.48-1.66)	0.88 (0.68-1.15)	0.85 (0.64-1.13)	1.04 (0.90-1.21)	0.97 (0.83-1.14)	1.11 (0.99-1.25)	1.09 (0.97-1.24)
Postpartum hemorrhage	0.94 (0.49-1.82)	0.91 (0.44-1.92)	1.32 (1.05-1.67)	1.42 (1.12-1.81)	1.24 (1.08-1.42)	1.27 (1.10-1.46)	1.04 (0.94-1.15)	1.03 (0.92-1.14)
**Neonatal outcomes**	**Unadjusted relative risk (95% CI)**	**Adjusted[a] relative risk (95% CI)**	**Unadjusted relative risk (95% CI)**	**Adjusted[a] relative risk (95% CI)**	**Unadjusted relative risk (95% CI)**	**Adjusted[a] relative risk (95% CI)**	**Unadjusted relative risk (95% CI)**	**Adjusted[a] relative risk (95% CI)**
Perinatal death	1.46 (0.21-10.36)	1.70 (0.24-12.09)	1.67 (0.70-4.03)	1.51 (0.56-4.03)	0.93 (0.48-1.78)	1.04 (0.54-2.00)	0.84 (0.51-1.38)	0.75 (0.42-1.32)
Preterm birth	1.53 (0.89-2.63)	1.06 (0.53-2.12)	1.42 (1.10-1.83)	1.34 (1.02-1.76)	1.44 (1.23-1.68)	1.35 (1.14-1.59)	1.41 (1.26-1.58)	1.31 (1.16-1.48)
Small for gestational age	1.55 (0.95-2.53)	1.19 (0.68-2.10)	1.19 (0.93-1.52)	1.15 (0.89-1.49)	1.08 (0.92-1.26)	1.01 (0.86-1.19)	1.05 (0.95-1.17)	1.08 (0.97-1.21)
Low birth weight (<2500 g)	1.18 (0.53-2.63)	0.63 (0.20-1.94)	1.39 (1.01-1.92)	1.33 (0.95-1.88)	1.40 (1.16-1.71)	1.26 (1.02-1.55)	1.35 (1.18-1.55)	1.29 (1.11-1.50)
Low Apgar score at 5 minutes	0.99 (0.32-3.09)	1.18 (0.38-3.65)	2.03 (1.39-2.96)	1.69 (1.10-2.60)	1.61 (1.25-2.07)	1.40 (1.05-1.85)	1.69 (1.44-1.98)	1.72 (1.45-2.02)
Neonatal hypoglycemia	1.99 (1.07-3.70)	1.29 (0.58-2.87)	0.87 (0.55-1.36)	0.73 (0.45-1.20)	1.48 (1.19-1.85)	1.34 (1.06-1.69)	1.54 (1.32-1.80)	1.40 (1.19-1.65)
Neonatal jaundice	0.64 (0.27-1.55)	0.60 (0.22-1.59)	1.38 (1.07-1.78)	1.35 (1.03-1.77)	1.10 (0.91-1.32)	1.01 (0.83-1.23)	0.95 (0.83-1.09)	0.98 (0.85-1.14)
Neonatal infections	0.35 (0.05-2.47)	1.50 (0.67-3.33)	1.50 (0.95-2.38)	1.73 (1.27-2.35)	1.45 (1.07-1.97)	1.36 (0.99-1.87)	1.19 (0.95-1.50)	1.13 (0.89-1.44)
Neonatal respiratory distress	1.27 (0.57-2.82)	0.40 (0.06-2.82)	1.82 (1.36-2.42)	1.42 (0.87-2.32)	1.82 (1.54-2.15)	1.69 (1.42-2.01)	1.69 (1.51-1.88)	1.58 (1.41-1.78)
Major congenital malformation	0.84 (0.32-2.25)	0.75 (0.24-2.31)	1.16 (0.85-1.59)	1.03 (0.72-1.46)	1.13 (0.94-1.36)	1.07 (0.88-1.30)	1.06 (0.94-1.20)	1.03 (0.90-1.17)
Minor congenital malformation	1.48 (0.48-4.59)	1.78 (0.58-5.54)	1.42 (0.95-2.13)	1.38 (0.90-2.11)	1.09 (0.86-1.37)	1.02 (0.80-1.31)	1.10 (0.96-1.26)	1.03 (0.89-1.19)

© 2023 Fernández de la Cruz et al. *JAMA Network Open.*

[a]Adjusted for age at delivery, parity, place of birth, education level, cohabitation with a partner, BMI, pre-pregnancy diabetes, smoking during pregnancy, and year of delivery.

© 2023 Fernández de la Cruz et al. *JAMA Network Open.*

eTable 5. Frequencies of Study Outcomes According to Obsessive-Compulsive Disorder (OCD) Status (Sweden only) and Dispensation of SRIs During Pregnancy in Mothers With OCD in Sweden (from July 2005 to 2019) and in British Columbia, Canada (from 2000 to 2019)

	Sweden			British Columbia, Canada	
	**Mothers without OCD (n=1,604,483)**	**Mothers with OCD *not* on SRIs during pregnancy (n=5,053)**	**Mothers with OCD on SRIs during pregnancy (n=2,990)**	**Mothers with OCD *not* on SRIs during pregnancy (n=445)**	**Mothers with OCD on SRIs during pregnancy (n=1,896)**
**Pregnancy and delivery outcomes**	**n (%)**	**n (%)**	**n (%)**	**n (%)**	**n (%)**
Gestational diabetes					
Yes	23021 (1.4)	75 (1.5)	70 (2.3)	26 (5.8)	164 (8.6)
No	1581462 (98.6)	4978 (98.5)	2920 (97.7)	419 (94.2)	1732 (91.4)
Preeclampsia					
Yes	43510 (2.7)	157 (3.1)	120 (4.0)	≤5	45 (2.4)
No	1560973 (97.3)	4896 (96.9)	2870 (96.0)	441 (99.1)	1851 (97.6)
Maternal infection					
Yes	5158 (0.3)	19 (0.4)	15 (0.5)	6 (1.3)	31 (1.6)
No	1599325 (99.7)	5034 (99.6)	2975 (99.5)	439 (98.7)	1865 (98.4)
Antepartum hemorrhage / placental abruption					
Yes	7189 (0.4)	25 (0.5)	15 (0.5)	14 (3.1)	55 (2.9)
No	1597294 (99.6)	5028 (99.5)	2975 (99.5)	431 (96.9)	1841 (97.1)
Premature rupture of membranes					
Yes	7670 (0.5)	26 (0.5)	27 (0.9)	31 (7.0)	62 (3.3)
No	1596813 (99.5)	5027 (99.5)	2963 (99.1)	414 (93)	1834 (96.7)
Induction of labor					
Yes	233266 (14.5)	940 (18.6)	532 (17.8)	89 (20.0)	479 (25.3)
No	1365985 (85.1)	4103 (81.2)	2455 (82.1)	356 (80.0)	1417 (74.7)
Missing	5,232 (0.3)	10 (0.2)	3 (0.1)	0	0
Mode of delivery					
Elective cesarean section	138141 (8.6)	592 (11.7)	391 (13.1)	39 (8.8)	214 (11.3)
Emergency cesarean section	128598 (8.0)	444 (8.8)	319 (10.7)	83 (18.7)	441 (23.3)
Instrumental delivery	107246 (6.7)	306 (6.1)	194 (6.5)	39 (8.8)	201 (10.6)
Vaginal delivery	1225266 (76.4)	3701 (73.2)	2083 (69.7)	284 (63.8)	1040 (54.9)
Missing	5232 (0.3)	10 (0.2)	≤5	0	0
Postpartum hemorrhage					
Yes	111475 (6.9)	360 (7.1)	281 (9.4)	40 (9.0)	156 (8.2)
No	1493008 (93.1)	4693 (92.9)	2709 (90.6)	405 (91.0)	1740 (91.8)

© 2023 Fernández de la Cruz et al. *JAMA Network Open.*

Neonatal outcomes	Live offspring of mothers without OCD (n=1,599,024) n (%)	Live offspring of mothers with OCD *not* on SRIs during pregnancy (n=5,039) n (%)	Live offspring of mothers with OCD on SRIs during pregnancy (n=2,975) n (%)	Live offspring of mothers with OCD *not* on SRIs during pregnancy (n=444) n (%)	Live offspring of mothers with OCD on SRIs during pregnancy (n=1,892) n (%)
Perinatal death[a]					
Yes	6165 (0.4)	16 (0.3)	14 (0.5)	≤5	7 (0.4)
No	1598318 (99.6)	5037 (99.7)	2976 (99.5)	444 (99.8)	1889 (99.6)
Preterm birth					
Yes	74788 (4.7)	284 (5.6)	240 (8.1)	53 (11.9)	236 (12.5)
No	1524236 (95.3)	4755 (94.4)	2735 (91.9)	391 (88.1)	1656 (87.5)
Small for gestational age					
Yes	104131 (6.5)	361 (7.2)	203 (6.8)	8 (1.8)	50 (2.6)
No	1492198 (93.3)	4667 (92.6)	2768 (93)	436 (98.2)	1842 (97.4)
Missing	2695 (0.2)	11 (0.2)	≤5	0	0
Low birth weight (<2500 g)					
Yes	49182 (3.1)	191 (3.8)	149 (5)	25 (5.6)	112 (5.9)
No	1547977 (96.8)	4840 (96.1)	2824 (94.9)	419 (94.4)	1780 (94.1)
Missing	1865 (0.1)	8 (0.2)	≤5	0	0
Low Apgar score at 5 minutes					
Yes	27492 (1.7)	94 (1.9)	143 (4.8)	13 (2.9)	118 (6.2)
No	1571532 (98.3)	4945 (98.1)	2832 (95.2)	430 (96.8)	1770 (93.6)
Missing	0	0	0	≤5	≤5
Neonatal hypoglycemia					
Yes	36878 (2.3)	136 (2.7)	124 (4.2)	6 (1.4)	42 (2.2)
No	1561874 (97.7)	4903 (97.3)	2851 (95.8)	438 (98.6)	1850 (97.8)
Neonatal jaundice					
Yes	73743 (4.6)	228 (4.5)	151 (5.1)	30 (6.8)	161 (8.5)
No	1525009 (95.4)	4811 (95.5)	2824 (94.9)	414 (93.2)	1731 (91.5)
Neonatal respiratory distress					
Yes	55606 (3.5)	238 (4.7)	265 (8.9)	36 (8.1)	186 (9.8)
No	1543146 (96.5)	4801 (95.3)	2710 (91.1)	408 (91.9)	1706 (90.2)
Neonatal infections					
Yes	20656 (1.3)	69 (1.4)	63 (2.1)	≤5	15 (0.8)
No	178096 (98.7)	4970 (98.6)	2912 (97.9)	441 (99.3)	1877 (99.2)
Congenital malformation					
Major	71423 (4.5)	257 (5.1)	146 (4.9)	51 (11.5)	236 (12.5)

© 2023 Fernández de la Cruz et al. *JAMA Network Open.*

Minor	48368 (3.0)	191 (3.8)	108 (3.6)	10 (2.3)	67 (3.5)
No	1479233 (92.5)	4591 (91.1)	2721 (91.5)	383 (86.3)	1589 (84.0)

[a]Including stillbirths and neonatal deaths within the first 27 completed days of life. The denominator for perinatal death was all livebirth or stillbirth at 22 completed weeks or later (i.e., as in Pregnancy and delivery outcomes above) and the denominator for the remaining variables in the table was live births at 22 completed weeks or later.

Abbreviations: OCD, obsessive-compulsive disorder; SRI, selective reuptake inhibitors.

© 2023 Fernández de la Cruz et al. *JAMA Network Open.*

eTable 6. Pregnancy, Delivery, and Neonatal Outcomes in Pregnancies and Births to Mothers With Obsessive-Compulsive Disorder (OCD) That Were Not on Serotonin Reuptake Inhibitors During Pregnancy in Sweden (from July 2005 to 2019), Compared With Mothers Who Did Not Have OCD

	Sweden	
**Pregnancy and delivery outcomes**	**Unadjusted relative risk (95% CI)**	**Adjusted[a] relative risk (95% CI)**
Gestational diabetes	1.03 (0.82-1.30)	1.19 (0.94-1.50)
Preeclampsia	1.15 (0.98-1.34)	1.07 (0.91-1.26)
Maternal infection	1.17 (0.75-1.84)	1.05 (0.64-1.71)
Antepartum hemorrhage / placental abruption	1.09 (0.80-1.46)	1.12 (0.82-1.53)
Premature rupture of membranes	1.08 (0.73-1.58)	0.90 (0.57-1.41)
Induction of labor	1.28 (1.20-1.36)	1.15 (1.08-1.23)
Elective cesarean section	1.36 (1.26-1.48)	1.38 (1.27-1.51)
Emergency cesarean section	1.13 (1.03-1.24)	1.09 (0.99-1.21)
Instrumental delivery	0.95 (0.85-1.06)	0.99 (0.88-1.11)
Postpartum hemorrhage	1.03 (0.92-1.14)	1.03 (0.92-1.15)
**Neonatal outcomes**	**Unadjusted relative risk (95% CI)**	**Adjusted[a] relative risk (95% CI)**
Perinatal death	0.82 (0.50-1.35)	0.77 (0.45-1.33)
Preterm birth	1.21 (1.07-1.35)	1.14 (1.00-1.29)
Small for gestational age	1.10 (0.99-1.22)	1.07 (0.96-1.19)
Low birth weight (<2500 g)	1.23 (1.07-1.42)	1.17 (1.00-1.36)
Low Apgar score at 5 minutes	1.09 (0.89-1.33)	0.96 (0.77-1.20)
Neonatal hypoglycemia	1.17 (0.99-1.38)	1.11 (0.93-1.33)
Neonatal jaundice	0.98 (0.86-1.12)	1.00 (0.87-1.15)
Neonatal respiratory distress	1.36 (1.20-1.54)	1.23 (1.07-1.41)
Neonatal infections	1.06 (0.84-1.34)	1.02 (0.79-1.31)
Major congenital malformation	1.15 (1.02-1.30)	1.05 (0.92-1.20)
Minor congenital malformation	1.26 (1.09-1.45)	1.07 (0.92-1.25)

[a]Adjusted for age at delivery, parity, place of birth, education level, cohabitation with a partner, BMI, pre-pregnancy diabetes, smoking during pregnancy, and year of delivery.

© 2023 Fernández de la Cruz et al. *JAMA Network Open.*
