# Supplementary material for: Pregnancy, Delivery, and Neonatal Outcomes Associated With Maternal Obsessive-Compulsive Disorder: Two Cohort Studies in Sweden and British Columbia, Canada
Source: JAMA Netw Open. 2023 Jun 14;6(6):e2318212. doi: 10.1001/jamanetworkopen.2023.18212 (PMC10267772; doi:10.1001/jamanetworkopen.2023.18212)
Supplement: Supplement 2. — Data Sharing Statement [file jamanetwopen-e2318212-s002.pdf]

Data Sharing Statement

Fernández de la Cruz. Pregnancy, Delivery, and Neonatal Outcomes Associated With Maternal Obsessive-Compulsive Disorder. *JAMA Netw Open*. Published June 14, 2023. doi:10.1001/jamanetworkopen.2023.18212

Data available: No
